# Emerging knowledge of the organelle outer membranes – research snapshots and an updated list of the chloroplast outer envelope proteins

**DOI:** 10.3389/fpls.2015.00278

**Published:** 2015-04-30

**Authors:** Kentaro Inoue

**Affiliations:** Department of Plant Sciences, University of California at DavisDavis, CA, USA

**Keywords:** Arabidopsis, chloroplast, membrane proteins, mitochondria, outer membrane

Mitochondria and chloroplasts are two distinct organelles essential for plant viability. They evolved from prokaryotic endosymbionts and share a common ancestor with extant Gram-negative bacteria (Gray et al., 1999; Gould et al., 2008). Successful conversion of the free-living prokaryotes to the cytoplasmic organelles via endosymbiosis required conservation and adaptation of the outer membranes to the dramatic change of surroundings. In prokaryotes, the outer membrane serves as a physical barrier that protects cells from the extracellular environment and allows import of necessary nutrients, and also directly participates in interaction with other organisms (Nikaido, 2003). As part of the semi-autonomous organelles, by contrast, the outer membranes of mitochondria and chloroplasts have gained ability to participate in intracellular communication and organelle biogenesis, i.e., import and export of various ions and metabolites, import of nuclear-encoded proteins, various metabolic processes including the biosynthesis of membrane lipids, and division and movement of the organelles that require physical interaction with cytoplasmic components (Breuers et al., 2011; Inoue, 2011; Duncan et al., 2013). Our understanding of the organelle outer membranes have been advanced greatly in the last decade or so, and the last eight years have seen about a three-fold increase in the number of proteins identified or predicted to be in the chloroplast outer envelope of *Arabidopsis thaliana* (Arabidopsis) [total 117 proteins listed in Table 1; compare 34 proteins in Inoue (2007)]. This Research Topic is intended to provide snapshots of recent research on the organelle outer membranes. It collects seven original research, three review and two method articles, which can be divided into four groups according to the subjects – (1) outer membrane protein targeting, (2) functions, targeting and evolution of protein import components, (3) lipid metabolism, and (4) method development.

## 1. Protein targeting to the organelle outer membranes

All proteins identified so far in the organelle outer membranes are encoded in the nucleus (e.g., Table 1), and most of them use internal signals for targeting. This is distinct from the case for most nuclear-encoded proteins found inside the organelles: they are synthesized with N-terminal extensions, which are necessary and sufficient for proper targeting via the general pathway and cleaved upon import in the matrix (mitochondria) or stroma (chloroplasts). Lee et al. (2014) review the current knowledge of pathways and signals needed for targeting of three types of outer membrane proteins – signal-anchored (SA), tail-anchored (TA), and β-barrel proteins. SA and TA proteins are anchored to the membrane via a single transmembrane (TM) α-helix with either N_intermembrane space_-C_cytosol_ (for SA) or N_cytosol_-C_intermembrane space_ (for TA) orientation. β-Barrel proteins are integrated into the membrane via multiple TM-β-strands, whose formation appears to require evolutionarily conserved machinery in the membrane. Marty et al. (2014) have used a transient expression system with *Nicotiana tabacum* Bright Yellow-2 suspension cells to identify two types of targeting signals for mitochondria TA proteins. They have then performed database search, increasing the number of mitochondria TA proteins from 20 to 54. Interestingly, 16 of the mitochondria outer membrane proteins identified by the previous work (Duncan et al., 2013) and Marty et al. (2014) are also found in the chloroplast outer envelope membrane (Table 1). This may suggest the presence of targeting mechanisms and functions shared between the outer membranes of the two organelles.

**Table 1:** **One hundred and seventeen proteins identified or predicted to be in the outer membrane of the Arabidopsis chloroplast envelope**.^a^

**AGI no.^b^**	**Name**	**References^c^**	**Envelope^d^**	**MitoOM^e^**
**SOLUTE/ION TRANSPORT**				
At1g20816	OEP21-1	(i)(ii)(iii)	YES	
At1g45170	OEP24-1	(i)(ii)(iii)(iv)		
At1g76405	OEP21-2	(i)(ii)(iv)	YES	
At2g01320	WBC7	(ii)(iii)(iv)	YES	
At2g17695	OEP23/DUF1990	(vii)	YES	
At2g28900	OEP16-1	(i)(ii)(iii)(iv)	YES	
At2g43950	OEP37	(i)(ii)(iii)(iv)	YES	
At3g51870	PAPST1 homolog	(viii)	YES	
At3g62880	OEP16-4	(i)(ii)		
At4g16160	OEP16-2	(i)(ii)		
At5g42960	OEP24-2	(i)(ii)	YES	
**PROTEIN IMPORT COMPONENTS AND THEIR HOMOLOGS**				
At1g02280	Toc33	(i)(ii)	YES	
At2g16640	Toc132	(i)(ii)(iii)(iv)	YES	
At2g17390	AKR2B	(iii)		
At3g16620	Toc120	(i)(ii)(iii)(iv)		
At3g17970	Toc64-III	(i)(ii)(iii)(iv)	YES	
At3g44160	P39/OEP80tr1	(ii)		
At3g46740	Toc75-III	(i)(ii)(iii)(iv)	YES	
At3g48620	P36/OEP80tr2	(ii)		
At4g02510	Toc159	(i)(ii)(iii)(iv)	YES	
At4g09080	Toc75-IV	(i)(ii)		
At5g05000	Toc34	(i)(ii)(iii)(iv)	YES	
At5g19620	OEP80/Toc75-V	(i)(ii)(iv)	YES	
At5g20300	Toc90	(i)(ii)(iv)		
**PROTEIN TURNOVER AND MODIFICATION**				
At1g02560	ClpP5 (proteolysis)	(iv)	YES	
At1g07930	E-Tu (protein synthesis)	(iii)		
At1g09340	HIP1.3/Rap38/CSP41B (protein synthesis)	(iv)	YES	
At1g63900	SP1 (proteolysis)	(vi)		
At1g67690	M3 protease	(iv)		
At3g46780	pTAC16 (transcription)	(iv)	YES	
At4g05050	UBQ11 (proteolysis)	(iii)(iv)		
At4g32250	Tyrosine kinase	(iii)(iv)	YES	
At4g36650	pBrP (transcription)	(ix)		
At5g16870	PTH2 family (protein synthesis)	(iii)(iv)		(x)
At5g35210	PTM (transcription)	(ii)	YES	
At5g56730	peptidase M16 family	(iv)	YES	(xi)
**LIPID METABOLISM**					
At1g77590	LACS9	(i)(ii)(iii)(iv)	YES	
At2g11810	MGD3	(i)(ii)		
At2g27490	ATCOAE	(iii)(iv)	YES	
At2g38670	PECT1	(iv)		(x)
At3g06510	SFR2/GGGT	(ii)(iii)(iv)	YES	
At3g06960	TGD4	(ii)	YES	
At3g11670	DGD1	(i)(ii)		
At3g26070	PAP/FBN3a	(iv)	YES	
At3g63170	FAP1	(iii)	YES	
At4g00550	DGD2	(i)(ii)		
At4g15440	HPL homolg	(i)(ii)	YES	
At5g20410	MGD2	(i)(ii)		
**CARBOHYDRATE METABOLISM AND REGULATION**				
At1g12230	transaldolase	(iv)	YES	
At1g13900	PAP2	(v)		(x)(xi)
At2g19860	HXK2	(iv)		(x)
At4g29130	HXK1	(iii)(iv)	YES	(x)
**OTHER METABOLISM AND REGULATION**				
At1g34430	PDC E2	(iv)	YES	
At1g44170	ALDH3H1	(iv)		
At2g34590	PDC E1beta	(iv)	YES	
At2g47770	TSPO	(ii)		
At3g01500	beta CA1	(iv)	YES	
At3g16950	PDC E3	(iv)	YES	
At3g25860	PDC E2	(iv)	YES	
At3g27820	MDAR4	(iii)(iv)	YES	
At5g17770	CBR	(iii)(iv)		(x)
At5g23190	CYP86B1	(i)		
At5g25900	KO1/GA3	(ii)		
**INTRACELLULAR COMMUNICATION**				
At2g16070	PDV2 (division)	(i)(ii)(iii)	YES	
At2g20890	THF1/PSB29 (plasma membrane)	(i)	YES	
At3g25690	CHUP1 (actin-dependent movement))	(ii)	YES	
At5g53280	PDV1 (division)	(i)(ii)		
At5g58140	PHOT2 (actin-dependent movement)	(iii)(iv)	YES	
**FUNCTIONS/LOCATIONS DEFINED IN COMPARTMENTS**				
**OTHER THAN THE CHLOROPLAST OUTER ENVELOPE**				
At1g27390	Tom20-2 (mito)	(iii)		(x)(xi)
At3g01280	VDAC1 (mito)	(i)	YES	(x)
At3g12580	Hsc70-4 (cytosol)	(iv)		
At3g21865	PEX22 (peroxisome)	(iv)		
At3g46030	histone H2B (nucleus)	(iii)		
At3g63150	MIRO2 (mito)	(iv)		(x)(xi)
At4g14430	enoyl-CoA isomerase (peroxisome)	(iii)		
At4g16450	Complex I subunit (mito)	(iii)		
At4g31780	MGD1 (IEM)	(iii)	YES	
At4g35000	APX3 (peroxisome)	(iii)(iv)	YES	(xi)
At4g38920	vacuolar ATPase sub	(iii)		
At5g02500	HSC70-1 (cytosol/nucleus)	(iv)	YES	
At5g06290	Prx B (stroma)	(iv)	YES	
At5g15090	VDAC3 (mito)	(i)	YES	(x)
At5g27540	EMB2473/MIRO1 (mito)	(iv)		(x)(xi)
At5g35360	CAC2/BC (IEM)	(iv)	YES	
**FUNCTIONS UNKNOWN/UNCLEAR**				
At1g09920		(iii)		(xi)
At1g16000	OEP9	(ii)		
At1g27300		(iii)		
At1g64850		(iv)	YES	
At1g68680		(iii)	YES	
At1g70480	DUF220	(iii)(iv)		
At1g80890	OEP9.2	(ii)		
At2g06010		(iv)		
At2g24440		(iii)		
At2g32240	DUF869	(iii)(iv)		(xi)
At2g32650	PTAC18 like	(iv)		
At2g44640		(iii)	YES	
At3g26740	CCL	(iii)		
At3g49350		(iii)		
At3g52230	OMP24 homolog	(i)(ii)(iii)	YES	
At3g52420	OEP7	(i)(ii)		
At3g53560	TPR protein	(iii)	YES	
At3g63160	OEP6	(ii)	YES	
At4g02482	putative GTPase	(ii)		
At4g15810	NTPase	(ii)		
At4g17170	RAB2	(iv)	YES	
At4g27680	NTPase	(iii)(iv)		
At4g27990	YGGT-B protein	(iii)	YES	
At5g11560		(iv)		
At5g20520	WAV2	(iv)		(x)
At5g21920	YGGT-A	(iii)		
At5g21990	OEP61-TPR	(ii)		
At5g27330		(iii)		
At5g42070		(iv)	YES	
At5g43070	WPP1	(iii)		
At5g51020	CRL	(ii)(iii)(iv)	YES	
At5g59840	RAB8A-like	(iv)		
At5g64816		(iii)	YES	

a *Names and functional categories are based on literatures cited in this work and databases. See Supplementary Material Table S1 for the extended name (if any), the location curated by various databases, and other predicted properties based on the primary sequence for each protein*.

b *Arabidopsis gene identifier (AGI) number, which represents the systematic designation given to each locus, gene, and its corresponding protein product by The Arabidopsis Information Resource (TAIR: https://www.arabidopsis.org/)*.

c *This list includes in total 117 proteins from two earlier review articles [32 from (i) Inoue (2007) and 44 from (ii) Breuers et al. (2011)], two recent chloroplast outer envelope proteomics studies [50 from (iii) Simm et al. (2013) and 58 from (iv) Gutierrez-Carbonell et al. (2014),] and five reports on individual outer envelope proteins [(v) PAP2 by Sun et al. (2012), (vi) SP1 by Ling et al. (2012), (vii) OEP23 by Goetze et al. (2015), (viii) PAPST1 by Xu et al. (2013), and (ix) pBrP by Lagrange et al. (2003)]. Note that Gigolashvili et al. (2012) predicts inner-envelope localization of PAPST1, and that the AGI number for pBrP was updated from At4g36655*.

d *YES indicates that the given protein was found in the chloroplast envelope proteomic studies (Ferro et al., 2003, 2010; Froehlich et al., 2003), which are listed in The Plant Proteome Database (PPDB: http://ppdb.tc.cornell.edu/) (Sun et al., 2009)*.

e *Proteins found in the mitochondrial outer membrane by (x) Duncan et al. (2013) or (xi) Marty et al. (2014)*.

## 2. Functions, targeting and evolution of protein import components

The most-studied chloroplast outer membrane proteins are subunits of the TOC (translocon at the outer-envelope-membrane of chloroplasts) machinery, which catalyzes the general pathway to import nuclear-encoded precursor proteins from the cytosol. Among the TOC components are homologous GTPases Toc159 and Toc34, which recognize the precursors and regulate their import, and Toc75, which forms a protein conducting channel. In Arabidopsis, there are four Toc159 isoforms which show substrate selectivity, two catalytically redundant Toc34 isoforms, and one functional Toc75 encoded on chromosome III (Table 1). Demarsy et al. (2014) review the current knowledge about how these subunits function and regulate protein import. Richardson et al. (2014) summarize available results and discuss functions, targeting and assembly of TOC subunits. Importantly, both review articles recognize outstanding questions about the TOC components, including the mechanisms of precursor recognition and their insertion into the membrane. By biochemical assays using chloroplasts isolated from pea seedlings, radiolabeled precursor proteins and recombinant proteins, Chang et al. (2014) demonstrate interaction of Toc159 isoforms called Toc132/Toc120 with a chloroplast superoxide dismutase (FSD1) that was predicted to comprise an exceptionally short import signal but has been shown otherwise, and also map the interaction domains beyond the N terminus. The interaction of FSD1 with Toc132, but not with Toc159, was also demonstrated by a split-ubiquitin yeast two-hybrid assay (Dutta et al., 2014). Grimmer et al. (2014) have used an *in vivo* approach, transiently producing GFP-tagged proteins in protoplasts of various Arabidopsis mutants and determining their N-terminal sequences by mass spectrometry analyses, and demonstrate that a plastid RNA binding protein is a substrate of Toc159. The Arabidopsis protoplast transient expression assay has also been used to define sequences required for targeting and membrane integration of a Toc159 ortholog (Lung et al., 2014). A previous genetic screening had demonstrated that Toc132 and Toc75 enhance root gravitropism signal transduction (Stanga et al., 2009). Strohm et al. (2014) now provide evidence supporting the involvement of plastids, instead of direct participation of TOC subunits, in the gravitropism signal transduction. Finally, Day et al. (2014) report phylogenetic relationships and *in vitro* targeting of the Toc75 homologs including the truncated forms of OEP80/Toc75-V, which are also known as P39 (Hsueh et al., 2014) and P36 (Nicolaisen et al., 2015) (Table 1).

## 3. Lipid metabolism

Under phosphate starvation, phospholipids in the cell membranes, mainly those in extraplastidic compartments, are used as the source of free phosphates and substituted by galactolipids made in the chloroplast outer envelope. Murakawa et al. (2014) have used Arabidopsis mutants and feeding assays to show that the outer-envelope-dependent galactolipid synthesis is stimulated by sucrose supplementation and this stimulation in turn enhances utilization of the added sucrose for plant growth. This work nicely illustrates the physiological significance of the metabolic activity localized in the chloroplast outer envelope for plant growth and development.

## 4. Method development

Hardre et al. (2014) report an attempt to apply biotin tagging and proteolysis to examine topology and membrane association of proteins in the spinach chloroplast. Although the work requires further refinement to achieve the desired specificity, the idea behind this approach is quite interesting. The *toc159*-null mutant is seedling-lethal thus has been examined as progenies of heterozygous parents. Tada et al. (2014) have established a method using Ziploc® container to grow the homozygous *toc159* mutants on the sucrose-supplemented media to the point that viable seeds can be obtained. This cost-effective method should be useful to study not only the *toc159*-null plant but also other recessive lethal mutants of photosynthesis.

In summary, the collection highlights various questions about the organelle outer membranes and interdisciplinary approaches employed to address them. The future research should use these and other strategies to answer questions about the proteins of known functions, in particular those involved in protein homeostasis, as well as those of unknown functions (Table 1). The editor greatly acknowledges the excellent contributions of all the authors and constructive comments by expert reviewers to each of the articles.

### Conflict of interest statement

The author declares that the research was conducted in the absence of any commercial or financial relationships that could be construed as a potential conflict of interest.

## Supplementary material

Table S1 **Extended names, curated locations and some other information of 117 proteins listed in Table 1**.

## References

[B1] Breuers F. K. Brautigam A. Weber A. P. (2011). The plastid outer envelope – a highly dynamic interface between plastid and cytoplasm. Front. Plant Sci. 2:97. 10.3389/fpls.2011.00097 22629266 PMC3355566

[B2] Chang W. Soll J. Bolter B. (2014). A new member of the psToc159 family contributes to distinct protein targeting pathways in pea chloroplasts. Front. Plant Sci. 5:239. 10.3389/fpls.2014.00239 24904628 PMC4036074

[B3] Day P. M. Potter D. Inoue K. (2014). Evolution and targeting of Omp85 homologs in the chloroplast outer envelope membrane. Front. Plant Sci. 5:535. 10.3389/fpls.2014.00535 25352854 PMC4195282

[B4] Demarsy E. Lakshmanan A. M. Kessler F. (2014). Border control: selectivity of chloroplast protein import and regulation at the TOC-complex. Front. Plant Sci. 5:483. 10.3389/fpls.2014.00483 25278954 PMC4166117

[B5] Duncan O. van der Merwe M. J. Daley D. O. Whelan J. (2013). The outer mitochondrial membrane in higher plants. Trends Plant Sci. 18, 207–217. 10.1016/j.tplants.2012.12.004 23291162

[B5a] Dutta S. Teresinski H. J. Smith M. D. (2014). A split-ubiquitin yeast two-hybrid screen to examine the substrate specificity of atToc159 and atToc132, two Arabidopsis chloroplast preprotein import receptors. PLoS ONE 9:e95026. 10.1371/journal.pone.0095026 24736607 PMC3988174

[B6] Ferro M. Brugiere S. Salvi D. Seigneurin-Berny D. Court M. Moyet L.. (2010). AT_CHLORO, a comprehensive chloroplast proteome database with subplastidial localization and curated information on envelope proteins. Mol. Cell. Proteomics 9, 1063–1084. 10.1074/mcp.M900325-MCP200 20061580 PMC2877971

[B7] Ferro M. Salvi D. Brugiere S. Miras S. Kowalski S. Louwagie M.. (2003). Proteomics of the chloroplast envelope membranes from *Arabidopsis thaliana*. Mol. Cell. Proteomics 2, 325–345. 10.1074/mcp.M300030-MCP200 12766230

[B8] Froehlich J. E. Wilkerson C. G. Ray W. K. McAndrew R. S. Osteryoung K. W. Gage D. A.. (2003). Proteomic study of the *Arabidopsis* thaliana chloroplastic envelope membrane utilizing alternatives to traditional two-dimensional electrophoresis. J. Proteome Res. 2, 413–425. 10.1021/pr034025j 12938931

[B9] Gigolashvili T. Geier M. Ashykhmina N. Frerigmann H. Wulfert S. Krueger S.. (2012). The Arabidopsis thylakoid ADP/ATP carrier TAAC has an additional role in supplying plastidic phosphoadenosine 5′-phosphosulfate to the cytosol. Plant Cell 24, 4187–4204. 10.1105/tpc.112.101964 23085732 PMC3517245

[B9a] Goetze T. A. Patil M. Jeshen I. Bolter B. Grahl S. Soll J. (2015). Oep23 forms an ion channel in the chloroplast outer envelope. BMC Plant Biol. 15:47. 10.1186/s12870-015-0445-1 25849634 PMC4331141

[B10] Gould S. B. Waller R. F. McFadden G. I. (2008). Plastid evolution. Annu. Rev. Plant Biol. 59, 491–517. 10.1146/annurev.arplant.59.032607.092915 18315522

[B11] Gray M. W. Burger G. Lang B. F. (1999). Mitochondrial evolution. Science 283, 1476–1481. 10.1126/science.283.5407.1476 10066161

[B12] Grimmer J. Rodiger A. Hoehenwarter W. Helm S. Baginsky S. (2014). The RNA-binding protein RNP29 is an unusual Toc159 transport substrate. Front. Plant Sci. 5:258. 10.3389/fpls.2014.00258 24982663 PMC4059279

[B12a] Gutierrez-Carbonell E. Takahashi D. Lattanzio G. Rodriguez-Celma J. Kehr J. Soll J.. (2014). The distinct functional roles of the inner and outer chloroplast envelope of Pea (Pisum sativum) as revealed by proteomic approaches. J. Proteome Res. 13, 2941–2953. 10.1021/pr500106s 24792535

[B13] Hardre H. Kuhn L. Albrieux C. Jouhet J. Michaud M. Seigneurin-Berny D.. (2014). The selective biotin tagging and thermolysin proteolysis of chloroplast outer envelope proteins reveals information on protein topology and association into complexes. Front. Plant Sci. 5:203. 10.3389/fpls.2014.00203 24999344 PMC4064156

[B14] Hsueh Y. C. Flinner N. Gross L. E. Haarmann R. Mirus O. Sommer M. S.. (2014). The chloroplast outer envelope protein P39 in *Arabidopsis thaliana* belongs to the Omp85 protein family. Proteins. [Epub ahead of print]. 10.1002/prot.24725 25401771

[B15] Inoue K. (2007). The chloroplast outer envelope membrane: The edge of light and excitement. J. Integr. Plant Biol. 49, 1100–1111 10.1111/j.1672-9072.2007.00543.x

[B16] Inoue K. (2011). Emerging roles of the chloroplast outer envelope membrane. Trends Plant Sci. 16, 550–557. 10.1016/j.tplants.2011.06.005 21775189

[B16a] Lagrange T. Hakimi M. A. Pontier D. Courtois F. Alcaraz J. P. Grunwald D.. (2003). Transcription factor IIB (TFIIB)-related protein (pBrp), a plant-specific member of the TFIIB-related protein family. Mol. Cell. Biol. 23, 3274–3286. 10.1128/MCB.23.9.3274-3286.2003 12697827 PMC153204

[B17] Lee J. Kim D. H. Hwang I. (2014). Specific targeting of proteins to outer envelope membranes of endosymbiotic organelles, chloroplasts, and mitochondria. Front. Plant Sci. 5:173. 10.3389/fpls.2014.00173 24808904 PMC4010795

[B17a] Ling Q. Huang W. Baldwin A. Jarvis P. (2012). Chloroplast biogenesis is regulated by direct action of the ubiquitin-proteasome system. Science 338, 655–659. 10.1126/science.1225053 23118188

[B18] Lung S. C. Smith M. D. Weston J. K. Gwynne W. Secord N. Chuong S. D. (2014). The C-terminus of Bienertia sinuspersici Toc159 contains essential elements for its targeting and anchorage to the chloroplast outer membrane. Front. Plant Sci. 5:722 10.3389/fpls.2014.00722 PMC4274882 25566294

[B19] Marty N. J. Teresinski H. J. Hwang Y. T. Clendening E. A. Gidda S. K. Sliwinska E.. (2014). New insights into the targeting of a subset of tail-anchored proteins to the outer mitochondrial membrane. Front. Plant Sci. 5, 426. 10.3389/fpls.2014.00426 25237314 PMC4154396

[B20] Murakawa M. Shimojima M. Shimomura Y. Kobayashi K. Awai K. Ohta H. (2014). Monogalactosyldiacylglycerol synthesis in the outer envelope membrane of chloroplasts is required for enhanced growth under sucrose supplementation. Front. Plant Sci. 5:280 10.3389/fpls.2014.00280 PMC4066442 25002864

[B21] Nicolaisen K. Missbach S. Hsueh Y. C. Ertel F. Fulgosi H. Sommer M. S.. (2015). The Omp85-type outer membrane protein p36 of *Arabidopsis thaliana* evolved by recent gene duplication. J. Plant Res. 128, 317–325. 10.1007/s10265-014-0693-4 25608613

[B22] Nikaido H. (2003). Molecular basis of bacterial outer membrane permeability revisited. Microbiol. Mol. Biol. Rev. 67, 593–656. 10.1128/MMBR.67.4.593-656.2003 14665678 PMC309051

[B23] Richardson L. G. Paila Y. D. Siman S. R. Chen Y. Smith M. D. Schnell D. J. (2014). Targeting and assembly of components of the TOC protein import complex at the chloroplast outer envelope membrane. Front. Plant Sci. 5:269. 10.3389/fpls.2014.00269 24966864 PMC4052903

[B23a] Simm S. Papasotiriou D. G. Ibrahim M. Leisegang M. S. Muller B. Schorge T.. (2013). Defining the core proteome of the chloroplast envelope membranes. Front. Plant Sci. 4:11. 10.3389/fpls.2013.00011 23390424 PMC3565376

[B24] Stanga J. P. Boonsirichai K. Sedbrook J. C. Otegui M. S. Masson P. H. (2009). A role for the TOC complex in Arabidopsis root gravitropism. Plant Physiol. 149, 1896–1905. 10.1104/pp.109.135301 19211693 PMC2663755

[B25] Strohm A. K. Barrett-Wilt G. A. Masson P. H. (2014). A functional TOC complex contributes to gravity signal transduction in Arabidopsis. Front. Plant Sci. 5:148. 10.3389/fpls.2014.00148 24795735 PMC4001062

[B25a] Sun F. Carrie C. Law S. Murcha M. W. Zhang R. Law Y. S.. (2012). AtPAP2 is a tail-anchored protein in the outer membrane of chloroplasts and mitochondria. Plant Signal. Behav. 7, 927–932. 10.4161/psb.20769 22751362 PMC3474687

[B26] Sun Q. Zybailov B. Majeran W. Friso G. Olinares P. D. van Wijk K. J. (2009). PPDB, the Plant Proteomics Database at Cornell. Nucleic Acids Res. 37, D969–D974. 10.1093/nar/gkn654 18832363 PMC2686560

[B27] Tada A. Adachi F. Kakizaki T. Inaba T. (2014). Production of viable seeds from the seedling lethal mutant ppi2-2 lacking the atToc159 chloroplast protein import receptor using plastic containers, and characterization of the homozygous mutant progeny. Front. Plant Sci. 5:243. 10.3389/fpls.2014.00243 24926298 PMC4045241

[B27a] Xu J. Yang J. Wu Z. Liu H. Huang F. Wu Y.. (2013). Identification of a dual-targeted protein belonging to the mitochondrial carrier family that is required for early leaf development in rice. Plant Physiol. 161, 2036–2048. 10.1104/pp.112.210831 23411694 PMC3613474

